# Development of high-performance aluminium 6061/SiC nanocomposites by ultrasonic aided rheo-squeeze casting method

**DOI:** 10.1016/j.ultsonch.2021.105631

**Published:** 2021-06-13

**Authors:** Thirugnanasambandam Arunkumar, T. Selvakumaran, Ram Subbiah, Karthikeyan Ramachandran, Sivakumar Manickam

**Affiliations:** a Department of Mechanical Engineering, CMR Institute of Technology, Bengaluru, India; b Department of Aerospace Engineering, SRM Institute of Science and Technology, Kattankulathur, Chennai, India; c Department of Mechanical Engineering, Gokaraju Rangaraju Institute of Engineering and Technology, Hyderabad, India; d Department of Aerospace and Aircraft Engineering, Kingston University London, UK; e Petroleum and Chemical Engineering, Faculty of Engineering, Universiti Teknologi Brunei, Bandar Seri Begawan BE1410, Brunei Darussalam

**Keywords:** Ultrasonication, Rheocasting, Al 6061/SiC, Grain refinement, Homogeneous distribution

## Abstract

In the modern era, the need for new products with novel processing and multipurpose materials is increased. The current market requirements for engineering applications are lightweight, high strength and low-cost materials. This paper explores the novel development process of high-performance nano cermet material for aerospace applications. Herein, lightweight aluminium 6061 + 2% of SiC (40 nm) nano cermet was fabricated through the casting method. The effects of ultrasonication, double stir casting or rheocasting, and squeezing pressure on nano cermet fabrication were successfully investigated by comparing their physical, thermal and mechanical properties. Scanning electron microscopy was employed to analyse the morphology of the cermets, and the presence of reinforcements was verified through EDS. The reinforcement of SiC into Al 6061 improved density, hardness, and reduction in porosity and grain refinement. This study reveals a novel fabrication process of ultrasonic-aided rheo-squeeze casting technique which enhanced the mechanical properties of the cermets compared to Al 6061 alloy due to nanoparticles homogeneous distribution, nominal agglomeration and porosity.

## Highlights

• Explored the ultrasonication aided novel fabrication process for Al/SiC cermet.
• This novel process enhanced the interfacing bonding between SiC & aluminium.
• SEM images confirm the nanoparticles homogeneous distribution & low-level porosity.

## 1 Introduction

Aluminium and its alloys are widely used in the aviation industry in the areas such as wings, rudder, exhaust pipes and fuselage due to its high strength to weight ratio, high creep and chemical resistance, load-bearing stability, rapid and low-cost manufacturing process [1]. Aluminium and its by-products are also used in military, marine, construction and automobile sectors [2], [3]. However, the excellent properties of Al-based materials at room temperature do not relate at elevated temperature, leading to the need for ceramic reinforcements for increasing their properties [4]. However, due to the mismatch of thermal expansion, weak bonding between ceramic and metal particles lead to undesirable degradation and thus lowered the thermal conductivity [5]. In recent years, researchers mostly prefer silicon carbide (SiC) as the reinforcement with aluminium in various applications such as cylinder liners, heat shields, space vehicles, high-performance brake pads due to its excellent properties that include high hardness, lower density and corrosion resistance [6]. Balaji *et al.* fabricated Al 7075-SiC metal matrix composite by a stir casting technique and reported that the hardness of the composite increased by 10% [7]. Likewise, 30% SiC reinforcement in Al 6061 through squeeze casting improved wear rate and enhanced mechanical properties [8]. As stated above, these metal matrix composites can be prepared by various methods such as casting, infiltration and powder metallurgy. Among these techniques, stir casting is a cheaper and simpler process that could form structures rapidly [9]. However, the development of high-performance nano cermets through this method is very challenging owing to the floating of nanoparticles on the molten liquid due to heterogeneous distribution, agglomeration and cluster formation [10]. To overcome this problem, double stir casting (rheocasting) strategy along with a vortex technique was combined to enhance the nanoparticle distribution [11]. However, the vortex method utilised higher stirring speed, allowing air to entrap which leads to porosity [12]. Hence, researchers have tried to control and further determine the ideal stirring speed and indicated that a lower stirring time leads to non-uniform distribution, whereas a higher stirring time leads to porosity [13]. Yang *et al.* introduced a novel ultrasonic vibration method to circumvent uniform distribution [14]. Using ultrasonication in stir casting improved the mechanical properties and microstructure refinement on the cermets [15], [16]. However, this technique failed to eliminate porosity due to external pressure [17]. Thus, external pressure is necessary for the squeeze casting process, which could compensate for the shortcoming of the double stir casting process by decreasing porosity along with increasing the mechanical properties of Al alloys [18], [19]. Similarly, squeeze casting led to the defect-free surface with improved toughness along with improved mechanical properties and grain refinement, leading to reduced porosity and cavities [20], [21].

In this context, the cermet of Al 6061/2% SiC was developed in this study using stir casting with different process combinations such as vortex, double stir casting (gravitational and squeeze) and ultrasonication, as illustrated in Table 1. The obtained cermet was then examined for its mechanical properties such as hardness, tensile strength and yield strength, and thermal conductivity as per ASTM standards. Overall, this investigation explored the development of a high-performance cermet through a novel strategy of ultrasonication-aided rheocasting.

**Table 1:** Fabrication process and sample notations of Al 6061 and its cermets.

Sample notations	Material	Fabrication process
AG	Aluminium 6061	Gravitational method
AS	Aluminium 6061	Squeeze method
ASDG	Aluminium 6061 + 2% SiC	Double stir casting - Gravitational method
ASDS	Aluminium 6061 + 2% SiC	Double stir casting - Squeeze method
ASDUG	Aluminium 6061 + 2% SiC	Double stir casting - Ultrasonic vibration - Gravitational method
ASDUS	Aluminium 6061 + 2% SiC	Double stir casting -Ultrasonic vibration - Squeeze method

## 2 Materials and methods

### 2.1 Materials

Commercially available Aluminium 6061 ingot according to ASTM B209 standards, magnesium and hexachloroethane were procured from Chemco Engineering Pvt. Ltd Chennai. Magnesium as a wetting agent and hexachloroethane (C_2_Cl_6_) as a degasser were used while fabricating the cermet. α-silicon carbide (SiC, 99%, particle size ~40 nm) was obtained from MK Industries, Canada [22].

### 2.2 Fabrication methodology

**Fig. 1:**
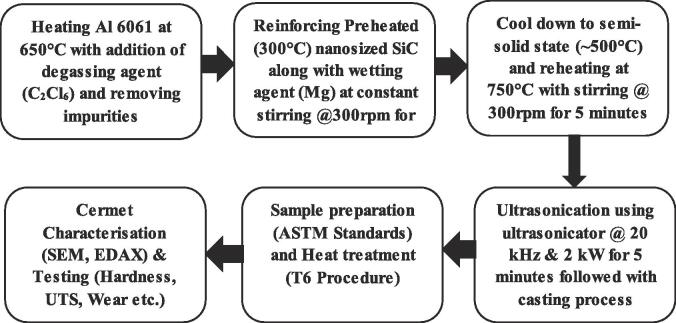
Schematic representing the novel fabrication process ultrasonication-aided rheocasting.

Aluminium 6061 base alloy and Al 6061 + 2% SiC cermets were fabricated with different process combinations, as shown in Table 1, employing a ceramics-aluminium stir casting machine. The following methodology, as shown in Fig. 1, has been adapted [23]. Initially, Al ingot was heated at 650 °C, and hexachloroethane (C_2_Cl_6_) was added into the molten liquid for degassing the alloy. Further, 2% SiC nanoparticles were preheated at 300 °C using an air oven and then added into the (650 °C)molten liquid at a constant stirring speed (300 rpm) using a mechanical impeller for 5 min along with Mg (wetting agent). The addition of degassing, wetting agent, and preheating of reinforcement removes the excess gases around the molten liquid alloy, thereby reducing oxidation and formation of external cavities. Further, the molten liquid was cooled into a semi-solid state at ~500 °C, to break down the clusters and reinforce agglomeration at lower viscosity. Again, the cooled liquid was heated to ~750 °C (above the melting point of Al 6061) at a constant stirring speed (300 rpm) for 5 min, allowing the nanoparticles to penetrate liquid metals along with improving homogeneous distribution [24].

Further to enhance homogeneity, an ultrasonication technique was carried out using titanium ultrasonic probe (M/s. Johnson Plastosonic, Pune, India) of dimensions 20 mm diameter and 200 length. The probe generated 20 kHz acoustic waves using 2 kW power and constant amplitude percentage and intensity of 40% and 43.72 W/cm^2^. The ultrasonic probe was immersed into the molten metal at approximately 2/3rd of the height and sonication was carried out for 5 min. The impact generated from the acoustics waves burst the clusters of Si nanoparticles on the molten metal. Subsequently, the liquid was poured onto the preheated steel die (~400 °C) of dimensions 150 × 150 × 50 mm using a gravitational method and squeeze method at 50 MPa for 3–6 min of solidification and then cooled to room temperature. The preheating of steel die avoids local stress concentration, which might lead to cracks through the surface. Finally, the solidified cermets were released and heat-treated following the T6 procedure, i.e., 530 °C for 5 h followed by quenching using cold water and ageing at 160 °C for 24 h to improve grain refinement and mechanical properties [25].

### 2.3 Sample preparation, characterisation and testing

The heat-treated samples (150 × 150 × 50 mm) were machined as per ASTM standards for carrying out various mechanical testing. The surface topographies of the samples were obtained using a scanning electron microscope (ZEISS SUPRA 55), and simultaneously quantitative elemental analysis was conducted as per ASTM D4541 [26], [27]. The density was measured using Archimedes method of liquid displacement using water as a liquid medium, and porosity (%) was calculated from the density [28]. Vickers hardness was measured using a diamond intender at a load of 10 N and a dwell time of 20 sec as per ASTM E92 (Vickers hardness machine, Krystal Elmec, India). Tensile test (UTM Zwick, Germany) was carried out as per ASTM E8/E8M-11 at a standard displacement of 0.5 mm/min. Wear behaviour of the samples were studied using a pin on disc tester (Ducom TR-20LE-PHM-400) as per ASTM G99 standards at a sliding speed of 2 m/s and varying load (20 N and 40 N) and sliding distance (600 m and 1200 m) [28], [29]. Thermal expansion (CLTE) was measured (Anter Unitherm Model-1161 V) under argon atmosphere as per ASTM E228, and thermal conductivity (TC) was measured (Netzsch LFA-467) as per ASTM E1225-13. To ensure adequate reproducibility of the findings, all the conducted experiments have been replicated three times.

## 3 Results and discussion

### 3.1 Morphology and elemental analysis

**Fig. 2:**
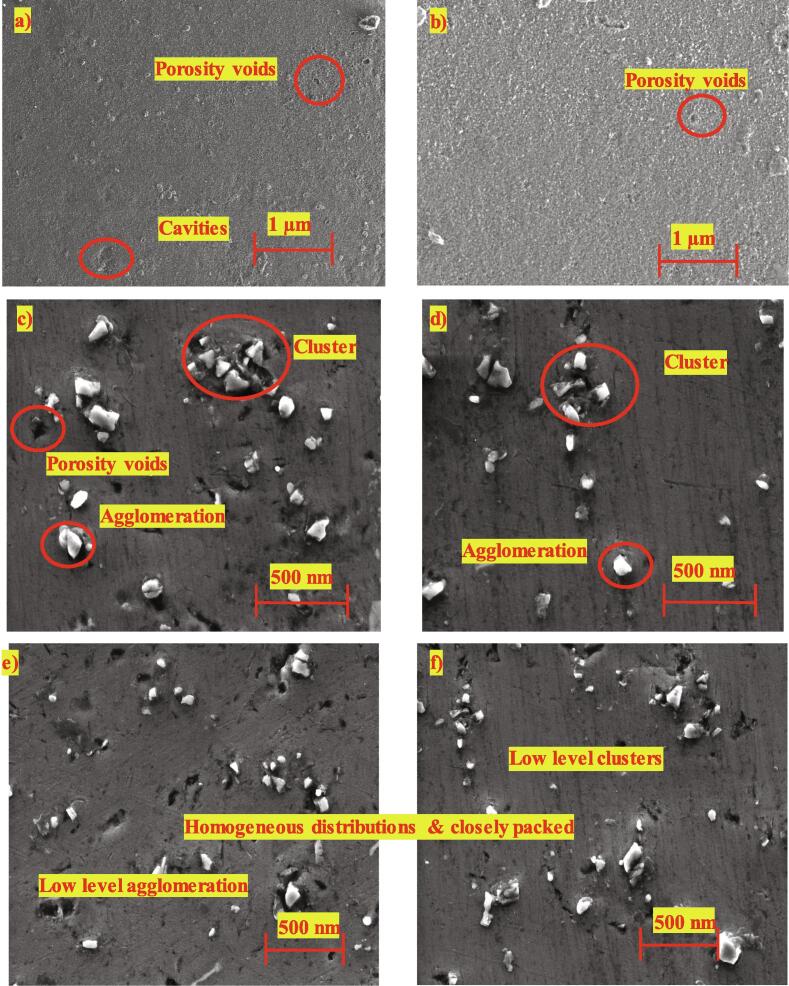
Scanning electron microscopic images of the cermets: a) AG b) AS c) ASDG d) ASDS e) ASDUG f) ASDUS.

Fig. 2 illustrates the scanning electron microscopic images of the Al 6061 along with nano-SiC reinforced cermet obtained through various fabrication techniques. The morphology of Al 6061 base material (Fig. 2a & b) indicates voids and cavities around the surface with reduced cavities in the squeeze technique. These voids in the Al 6061 could have been due to the followed fabrication process and heat treatment.

However, in the nano-SiC reinforced Al 6061 cermet, different fabrication processes were carried out, and morphology was studied and presented in Fig. 2(c) to 2(f). In the double casting process of reinforced cermets, the agglomeration of SiC nanoparticles could be noted along with porous voids on the surfaces of ASDG (Al 6061/2% SiC fabricated through double-casting & gravitational method. However, the squeeze-aided double-casting method reduced voids but with the same quantity of clusters and agglomeration in SiC. These studies exhibit that the squeeze technique reduces porosity on the surfaces of the cermets, as observed by the researchers [18], [30].

**Fig. 3:**
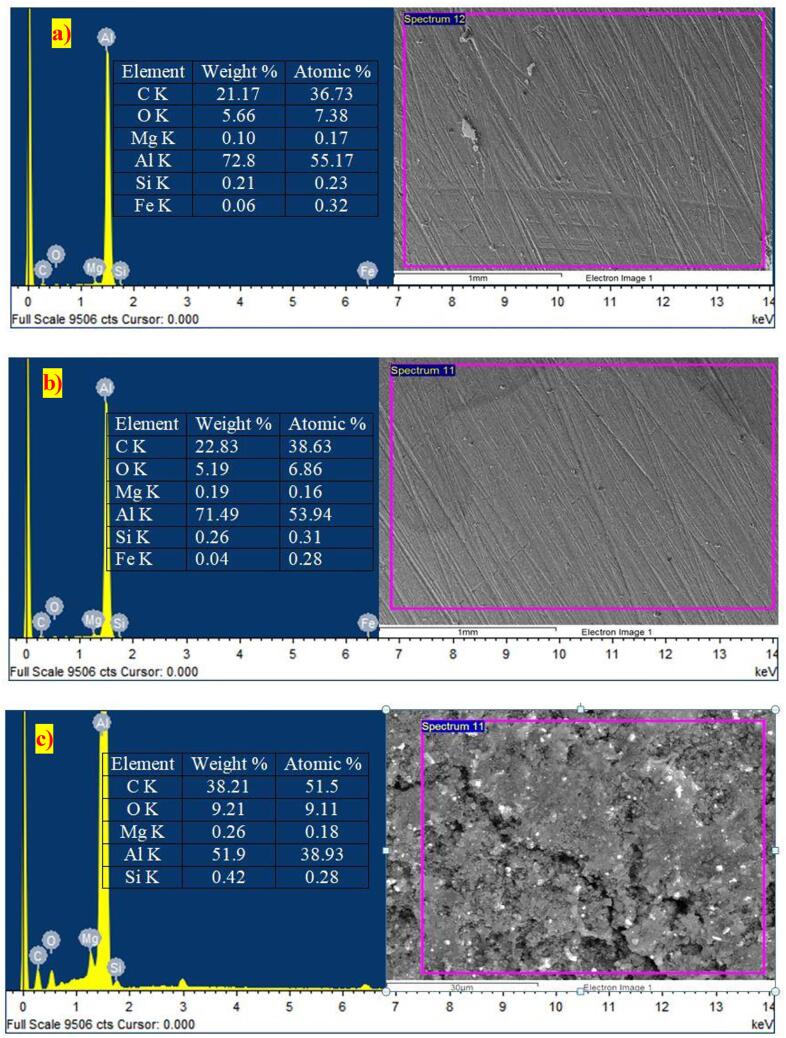

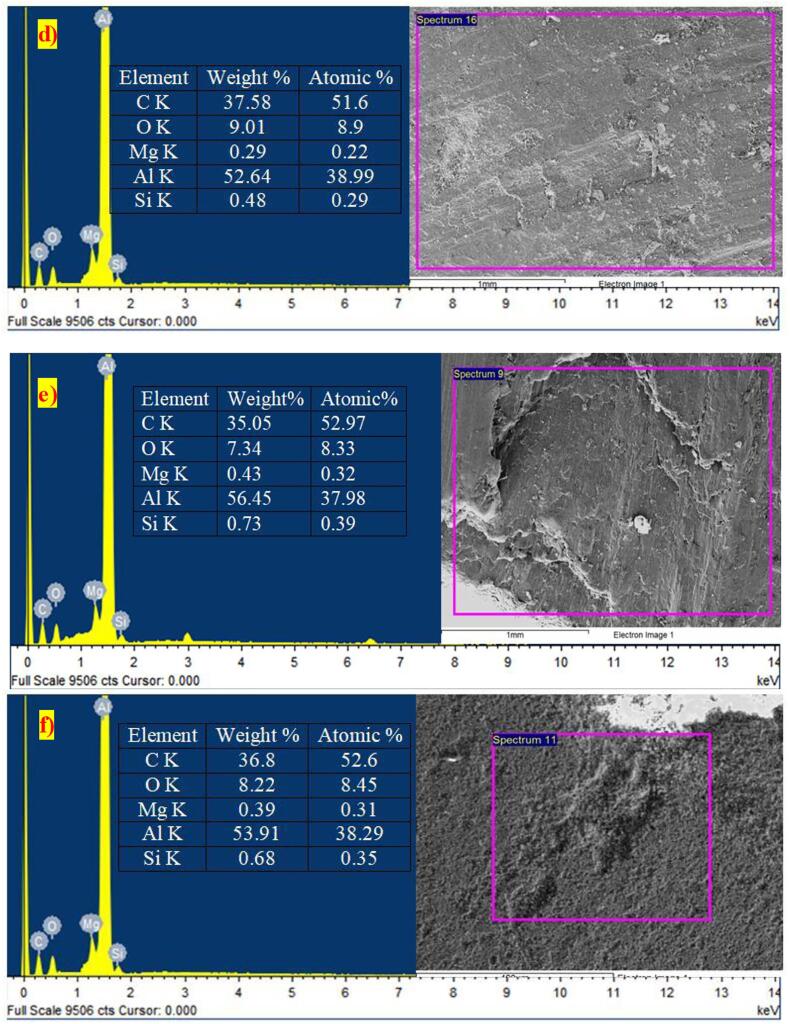
Elemental analysis (EDAX) of cermets: a) AG b) AS c) ASDG d) ASDS e) ASDUG f) ASDUS. Where C represents carbon, O represents oxygen, Mg represents magnesium, Al represents aluminium and Si represents silicon.

On the other hand, ultrasonication aided the rheo-squeeze casting process with gravitational (ASDUG) and squeeze method (ASDUS) demonstrated a higher distribution of nanoparticles with nominal agglomeration and clusters compared to cermets obtained by other processes. The observed higher distribution could have been due to mechanical stirring using an impeller at solid–liquid temperature (500 °C), which decreases the size of nanoparticle agglomeration due to higher friction between the semi-solid molten liquid. Also, the ultrasonication vibration creates acoustic waves that increase the uniform distribution of nanoparticles homogeneously in the melt at lower viscosity of molten liquid [23]. Among the many ceramic reinforcements considered for making aluminum matrix composites, Al_2_O_3_ and SiC have been found to have an excellent compatibility with the aluminum matrix [31] Since SiC offers an adequate thermal stability with aluminum alloy during the synthesis and application. Herein, the compatibility i.e bonding between the aluminium and SiC is significantly enhanced due to appropriate (Mg) wetting agent, reheating temperature (750 ^O^C), and additional squeeze pressure. Further, a quantitative analysis was carried out to verify the presence of SiC and Al 6061 throughout the surface. The elemental analysis (Fig. 3) shows clear peaks of Al, Si, C and Mg (wetting agent) throughout the cermets with higher grain refinements.

### 3.2 Porosity and density

**Fig. 4:**
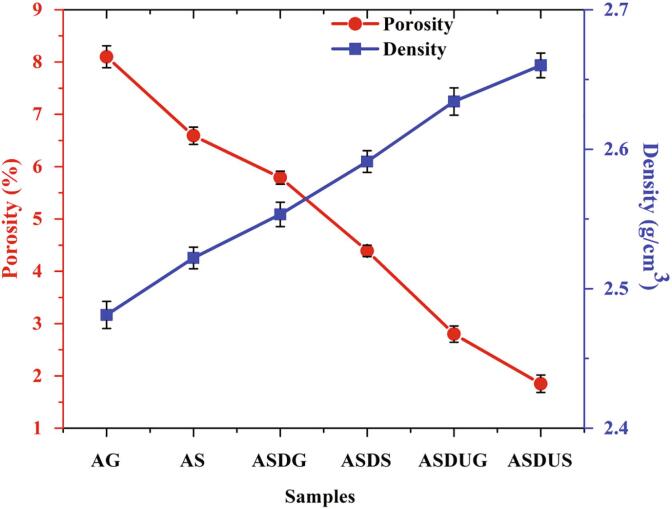
Archimedes density and porosity of the Al 6061 and its reinforced cermets.

Fig. 4 presents the density and porosity (%) of the Al 6061 and its cermets fabricated through different routes. The obtained experimental values are shown in Table 2, and it can be seen that the fabrication technique has a significant impact on the resultant density and porosity of the samples. Generally, with the addition of SiC ceramic, the density of the sample is expected to decrease based on the rules of mixtures [32]. However, in this scenario, a nominal gain in the density is noted, which could be due to the applied fabrication techniques associated with cermet development. The squeezing pressure applied during fabrication increased the AS, ASDS and ASUDS cermets compared to its counterparts. Further, particle dispersion also played a major role in the increased density [32]. The ultrasonication and degassing of the cermet before heat treatment led to uniform distribution of SiC throughout the base material, which enhances the density with a considerate reduction in porosity [33]. Moreover, in the squeeze casting process (ASDS), the cermet porosity decreased by 45.80%, and density increased by 4.43% relative to the base material. Besides, the porosity of ASDUS and ASDUG cermets drastically reduced by 57.85% and 51.64%, respectively, compared to ASDS and ASDG cermets due to ultrasonication. Similarly, the porosity of ASDUS and ASDS cermets reduced by 33.92% and 24.17%, respectively compared to ASDUG and ASDG due to squeezing pressure. From these results, it is evident that ultrasonication and squeeze pressure are the important parameters influencing the porosity and density of the cermets.

**Table 2:** Properties of Al 6061 and its cermets fabricated through different processes.

Samples	Density (g/cm^3^)	Hardness (MPa)	Avg. Grain size (µm)	Porosity (%)	UTS (MPa)	YS (MPa)	E (%)	TC (W/m.K)	CLTE (×10^-6^ /°C)
AG	2.4812	911.5	41.66	8.10	266.2	258.32	11.5	165	22.49
AS	2.5221	922.8	35.71	6.59	271.8	261.25	11.3	164	22.29
ASDG	2.5533	950.8	31.25	5.79	273.4	262.21	11.2	160	21.02
ASDS	2.5912	970.5	27.77	4.39	281.5	264.42	10.8	158	20.73
ASDUG	2.6342	1012	25.00	2.80	295.1	266.51	10.6	155	20.69
ASDUS	2.6601	1033	20.83	1.85	299.9	268.21	10.4	153	20.31

### 3.3 Hardness and grain size

**Fig. 5:**
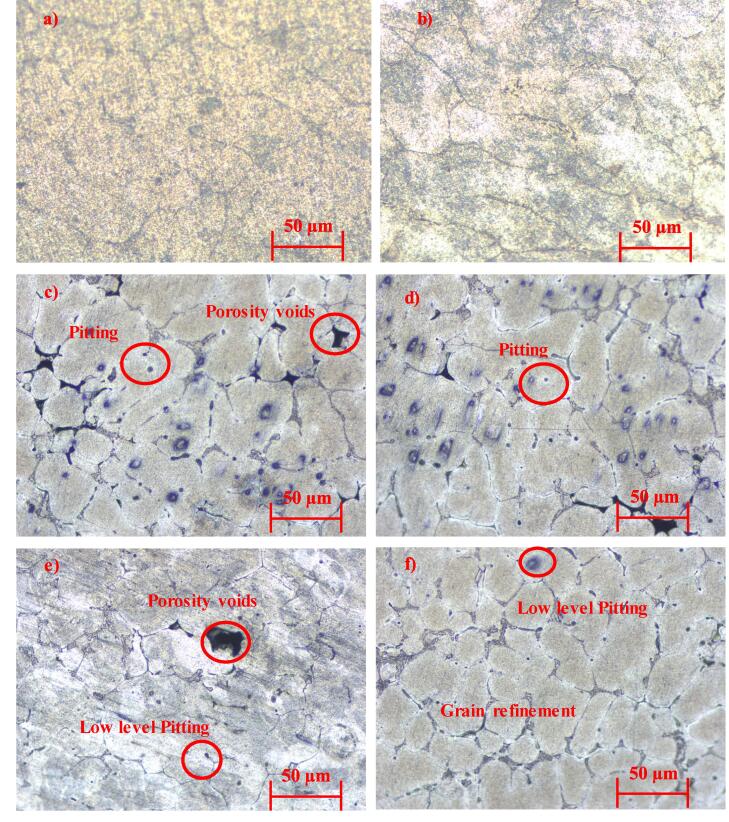
Microstructure of the samples: a) AG b) AS c) ASDG d) ASDS e) ASDUG f) ASDUS.

**Fig. 6:**
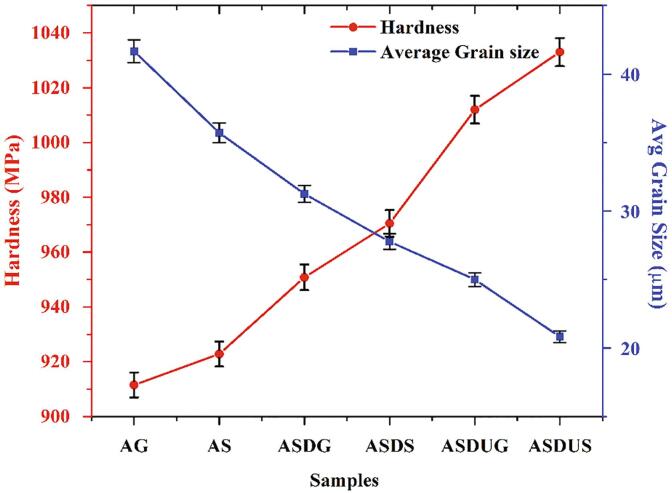
Vickers hardness and average grain size of the samples.

Fig. 5(a-f) illustrates the microstructure of the cermets fabricated through different processes. The average grain size of the samples was calculated through the line intercept method using the microstructural images with the help of ImageJ software. The microstructure of the cermets shows the presence of pitting and voids, as shown in Fig. 5 (c & d). In contrast, the ultrasonic-aided rheocasting led to finer grain refinement with a lower level of pitting and reduced porous voids (Fig. 5(f)) in the squeeze casting process. However, the gravitational method aided with ultrasonication still showed voids on the surfaces (Fig. 5(e)) due to the lack of external pressure. This presence is also visible in Fig. 5(c) where gravitational method led to increased porous structure. To overcome this issue, external force of 50 MPa for 3–6 min of solidification was used to reduce the voids in their novel fabrication process. The finer grain refinement could have been due to ultrasonication and squeezing pressure acting on the surface of the cermets [23]. The Vickers hardness of the samples increased with a reduction in grain size, as shown in Fig. 6. Besides, Wagih et al reported that the main strengthening mechanism is the grain refinement while the addition of SiC acts as a secondary strengthening source [34]. In the ultrasonic-assisted rheo-squeeze casting (ASDUS) process, the cermet grain size was reduced by 50%. In comparison, hardness was increased by 13.32% compared to base materials (AS) due to superior grain refinement. Similarly, in the rheo-squeeze casting method (ASDS), the cermet grain size was decreased by 33.34% relative to the base material, whereas the hardness was increased by 6.47%. Moreover, the hardness of cermet via ASDUS, ASDUG processes was increased by 6.43% compared to ASDS and ASDG processes due to the ultrasonication effect. On the other hand, in ASDUS, ASDS processes, hardness was increased by 2.07% compared to ASDUG and ASDG processes due to squeezing pressure. This can be due to influence of SiC, homogeneous distribution, strong bonding, and better refinement of the cermet through ultrasonic effect and squeeze pressure as reported earlier [23].

### 3.4 Tensile behaviour

**Fig. 7:**
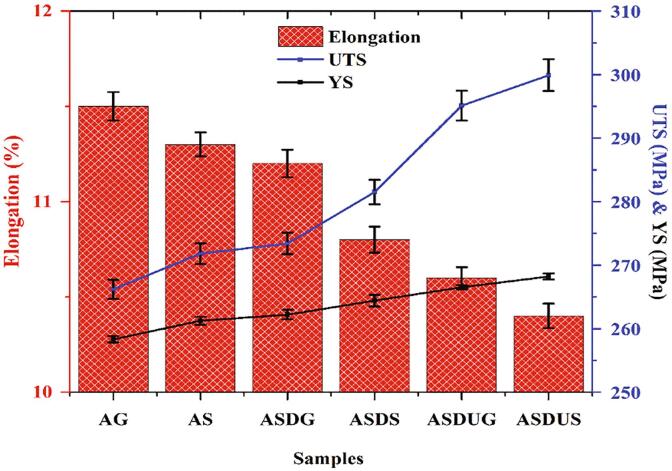
Ultimate tensile strength, yield strength and elongation of Al 6061 and its SiC reinforced cermets.

Ultimate tensile strength (UTS) and yield strength (YS), and elongation were calculated as per ASTM standards, as illustrated in Fig. 7. The UTS and YS increase with a nominal decrease in elongation due to different fabrication techniques. ASDS cermet showed an increase of 5.74% and 2.36% in UTS and YS, respectively, compared with Al 6061. Similarly, most of the cermets with SiC reinforcement exhibit an increase in UTS by 1.62%, 2.96%, 6.53% and 7.93% from ASDG, ASDS, ASDUG and ASDUS cermets, respectively. The tensile strength observed in this study is comparatively closer to reported values in the literature [35], [36], [37]. The tensile strength improvement is due to grain refinement and homogeneous distribution of SiC in aluminium matrix. It is well known SiC particulates is higher strength than aluminium. Also novel fabrication technique could improve the higher interfacial bonding between SiC and Al alloy and higher grain refinement. The acoustic waves homogeneously distribute the SiC nanoparticles throughout the cermets. The increase in tensile strength could also relate to the addition of SiC particles, increasing the strength by accelerating the bonding between the Al-SiC matrix [38], [39]. This fabrication on the cermets through varying parameters led to an increase in the strength with a reduction in elongation. This nominal decrease in the elongation could result from incorporating hard and brittle natured ceramics, which reduces the ductility of Al 6061 alloy leading to a reduction in elongation. Further, the addition of SiC into the Al matrix led to embrittling in the cermet, increasing the cermet's strength. Overall, the increase in tensile strength and decrease in elongation could be due to the employed fabrication techniques and ceramic reinforcement into the base materials.

### 3.5 Thermal behaviour

**Fig. 8:**
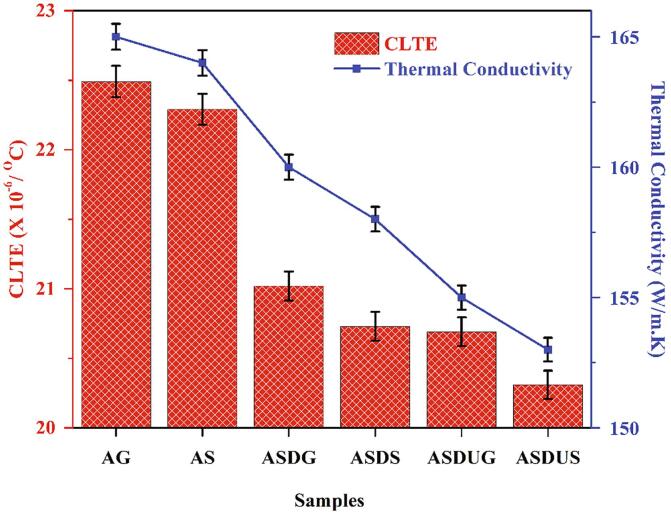
Thermal conductivity and coefficient of thermal expansion of Al 6061 and its cermets.

The Al 6061 and its SiC reinforced composites counterparts were studied for its thermal behaviour with help of thermal conductivity (TC) and expansion coefficient (CLTE). The thermal studies on the cermets were highly dependent of fabrication techniques and ceramic reinforcement. Various research has been carried out around the thermal conductivity and expansion coefficient studies and typically the incorporation of SiC or ceramic based reinforcement led to decrease in conductivity and expansion coefficient due to high thermal stability of ceramics [40]. The results of thermal studies are plotted in Fig. 8 where a steep decrease in the thermal conductivity and expansion values are being observed with change in fabrication method. ASDS cermets shown a decrease in TC and CLTE by 4.24% and 7.84%, respectively relative to the base material. Also, for ASDUS cermets TC and CLTE were reduced by 7.27% and 9.69%, respectively compared to Al 6061 (base material). This reduction in the thermal conductivity and expansion throughout the cermet in different fabrication method could be due to the solidification behaviour and interfacing bonding between the cermets. The base material of Al 6061 was fabricated through two different approaches likely to be gravitational method (AG) and squeeze casting (AS) which shown a slight decrease in the TC and CLTE value resulting that the applied external pressure was responsible as it increased the density while reducing the porosity. The squeeze casting method increased the solidification rate of the cermets which resulted in higher interfacing bonding between SiC particles and Al alloy. However in rheo squeeze casted cermets (ASDG and ASDS), reduction in the TC and CLTE were noted to be to higher than AG and AS due to the higher interfacing bonding and reduced porous gaps. This was also reported from the study by Safi *et al.* where they reported that density and porosity played a huge role in reduction of TC and CLTE [41]. Q Zhang *et al.* also supported the decrease in thermal conductivity with SiC additives into Al composites in their work [42]. In case of ultrasonic aided rheo squeeze casting technique (novel technique), the TC and CLTE of the cermets shown a higher reduction than the all-other fabrication techniques. This could be due to the even distribution of the nanoparticles through high acoustic waves emitted from the ultrasonic probe tool, this even distribution of the SiC particles enhance the density by increasing the interfacial bonding between Al matrix and SiC particles which resulted in reduction of porous voids and increase in density. The interfacing bonding between Al and SiC matrix could have played a crucial role as SiC bonding drastically result in the decrease of TC and CLTE. Our previous study on the novel technique also reported the same steep decrease due to the fabrication method [23].

### 3.6 Sliding wear

**Fig. 9:**
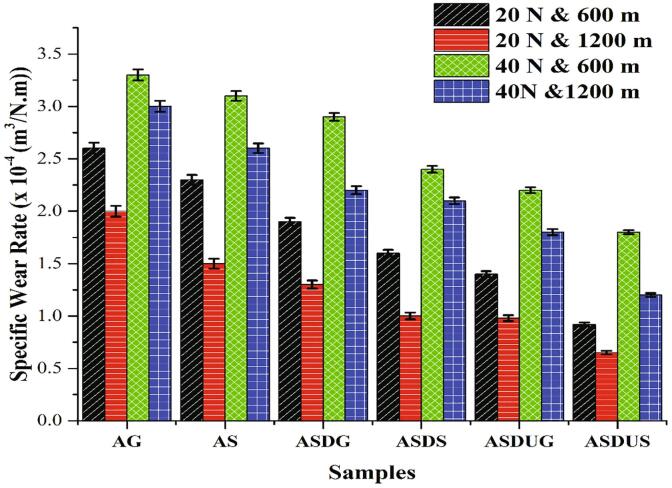
The specific wear rate of the samples with varying load and distance.

The specific wear rate (SWR) of the cermet and base material were calculated by varying load and sliding distance based on different fabrication processes, as shown in Fig. 9. The specific wear rate of the cermets decreased with SiC reinforcement and due to fabrication techniques. Likewise, wear resistance is enhanced by adding hard SiCparticles to the soft aluminium matrix [43], [44]. The SWR of the gravitational method in ASDG and ASDUG cermets shows a decrease of 29.62% and 46.15%, respectively compared to the base material (AG). Also, in the squeeze pressure process, ASDS and ASDUS cermets reduced by 44.47% and 60%, respectively compared to AS samples.

**Fig. 10:**
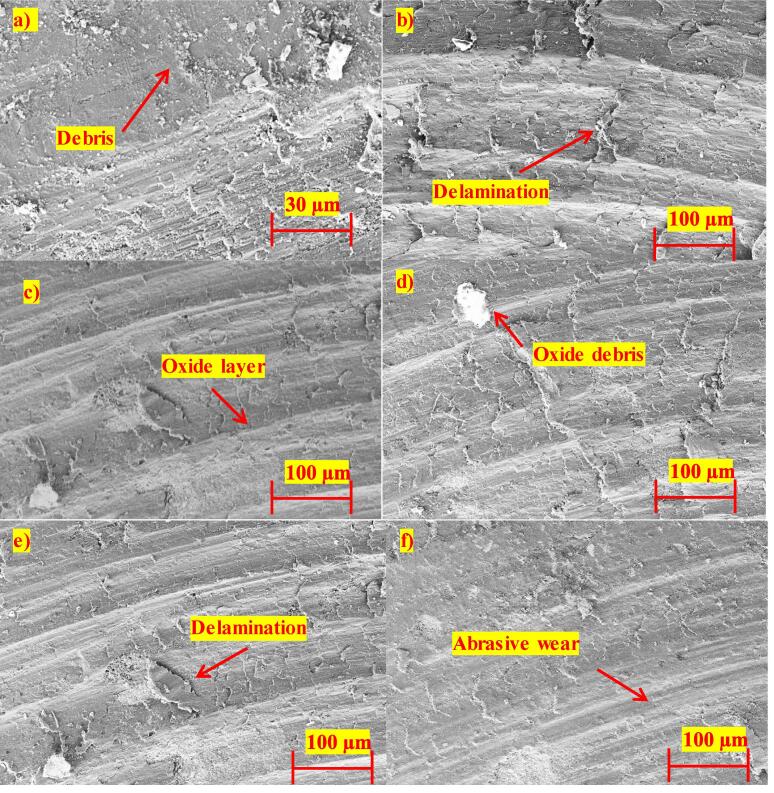
SEM images of wear worn surfaces of the samples: a) AG b) AS c) ASDG d) ASDS e) ASDUG f) ASDUS.

However, the novel fabrication technique with squeeze pressure and ultrasonication shows a drastic reduction of 65% SWR in the ASDUS cermet. The reduction in the wear rate at low load could be due to the formation of oxides on the surfaces when samples interact with the metal contacting surface. These formed oxides affect the tribological characteristics, leading to a lower wear rate [45]. When the load increases from 20 N to 40 N, the heat between the contacting surfaces increases and the oxide layers wear out, preventing them from forming additional layers when in contact with metal surfaces [46]. When sliding distance increases, the wear rate decreases by 29.34% (20 N) and 33.33% (40 N) due to the solid lubricant effect between the contact surfaces, pin and disc materials. This lubricating film acts as an insulator and does not allow any temperature rise to soften the pin material to undergo further wear. Besides, wear characteristics not only depending on the hardness of the material but also on whether self-protective mechanisms may be activated [47]. Fig. 10 shows that worn debris, scratch, cracks are high in the base material (AG and AS) and less in other reinforced samples (ASDG, ASDS, ASDUG, ASDUS). The existence of adequate nanoparticles is obvious since a tribo- mechanically oxide layer is developed between the composite pin and steel (disk), reducing the SWR. Therefore, it can be concluded that the proposed novel strategy is beneficial to fabricate ASDUS cermets in order to improve the wear resistance and make them suitable for aerospace applications.

## 4 Conclusion

Al 6061 alloy and its reinforced cermet with nanosized SiC were prepared through different fabrication processes. This study intends to bring out the effects of casting, rheocasting, squeezing pressure and ultrasonication processes associated with the physical, mechanical and thermal properties of the cermets. Further, a novel strategy of combining ultrasonication, rheocasting and squeeze pressure was also explored. The SiC reinforced cermets exhibited enhanced mechanical properties than Al 6061 base metal fabricated through different routes. The developed ASDUS cermet shows a significant increase in density (7.21%), hardness (13.32%), tensile strength (12.65%) and a satisfactory decrease in porosity (77.61%), thermal conductivity (7.27%) and thermal expansion coefficient (9.69%). The specific wear rate also showed a decrease of 80.3% than the base material owing to the formation of oxide scales throughout the surfaces. This shift in the material behaviour could be due to the employed fabrication process, which led to homogeneous distribution with a nominal level of nanoparticle clusters and enhanced grain refinement, as observed through the morphological study. These results evidence that the developed novel rheocasting aided with ultrasonication and squeeze pressure fabrication process impacts the Al-based metal matrix composites through enhancing the material behaviour by providing less porosity and homogenous distribution of nanoparticles along with lesser metal wastage.

## Declaration of Competing Interest

The authors declare that they have no known competing financial interests or personal relationships that could have appeared to influence the work reported in this paper.
